# Search for Allergens from the Pollen Proteome of Sunflower (*Helianthus annuus* L.): A Major Sensitizer for Respiratory Allergy Patients

**DOI:** 10.1371/journal.pone.0138992

**Published:** 2015-09-29

**Authors:** Nandini Ghosh, Gaurab Sircar, Bodhisattwa Saha, Naren Pandey, Swati Gupta Bhattacharya

**Affiliations:** 1 Division of Plant Biology, Bose Institute, Kolkata, West Bengal, India; 2 Department of Allergy and Asthma, Belle Vue Clinic, Kolkata, India; Henan Agricultural Univerisity, CHINA

## Abstract

**Background:**

Respiratory allergy triggered by pollen allergens is increasing at an alarming rate worldwide. Sunflower pollen is thought to be an important source of inhalant allergens. Present study aims to identify the prevalence of sunflower pollinosis among the Indian allergic population and characterizes the pollen allergens using immuno-proteomic tools.

**Methodology:**

Clinico-immunological tests were performed to understand the prevalence of sensitivity towards sunflower pollen among the atopic population. Sera from selected sunflower positive patients were used as probe to detect the IgE-reactive proteins from the one and two dimensional electrophoretic separated proteome of sunflower pollen. The antigenic nature of the sugar moiety of the glycoallergens was studied by meta-periodate modification of IgE-immunoblot. Finally, these allergens were identified by mass-spectrometry.

**Results:**

Prevalence of sunflower pollen sensitization was observed among 21% of the pollen allergic population and associated with elevated level of specific IgE and histamine in the sera of these patients. Immunoscreening of sunflower pollen proteome with patient sera detected seven IgE-reactive proteins with varying molecular weight and pI. Hierarchical clustering of 2D-immunoblot data highlighted three allergens characterized by a more frequent immuno-reactivity and increased levels of IgE antibodies in the sera of susceptible patients. These allergens were considered as the major allergens of sunflower pollen and were found to have their glycan moiety critical for inducing IgE response. Homology driven search of MS/MS data of these IgE-reactive proteins identified seven previously unreported allergens from sunflower pollen. Three major allergenic proteins were identified as two pectate lyases and a cysteine protease.

**Conclusion:**

Novelty of the present report is the identification of a panel of seven sunflower pollen allergens for the first time at immuno-biochemical and proteomic level, which substantiated the clinical evidence of sunflower allergy. Further purification and recombinant expression of these allergens will improve component-resolved diagnosis and therapy of pollen allergy.

## Introduction

Allergy is a type of abnormal immune reactions, which is triggered by environmental antigens or allergens and mediated by IgE antibodies. Pollen grains are the most common sources of inhalant allergens and responsible for inducing respiratory allergic disorders. Around 15–30% of world population was estimated to suffer from respiratory allergy caused by pollen grains of ambient outdoor environment [1]. Pollen allergen induced inflammation of respiratory tract is thought to be mediated by activation of two signaling pathways, one of which leads to ROS generation resulting in dendritic cell dysfunction and the other one disrupts the Th1/Th2 balance leading to enhancement of Th2 subpopulation [2]. Currently the mainstay in allergy treatment relies on the use of anti-histamines, non-steroidal anti-inflammatories and corticosteroids in acute cases, which may most often have severe side effects. The only disease modifying approach is thought to be allergen-specific immunotherapy in which the allergy inducing molecule i.e. the allergen itself is used for vaccination. Furthermore, the clinical diagnosis of allergy is also based on the use of purified allergens which is thought to be more accurate and sensitive than using crude pollen extract. Hence, proper identification and characterization of allergens are important in order to efficiently utilize the current diagnostic and therapeutic tools for allergy.

Some of the plants belonging to Asteraceae family such as *Ambrosia artemisiifolia* (Ragweed) [3, 4, 5], *Artemisia vulgaris* (mugwort) [5, 6, 7] and *Parthenium hysterophorus* [8, 9] are important sources of pollen allergens. Economically, the most important plant of this family (Asteraceae) is *Helianthus annuus* (sunflower), which is an agriculturally important oilseed crop and also an ornamental flowering plant. Although there are several clinical reports from different parts of the world on the occurrence of sunflower pollen allergy, still the knowledge has been restricted due to lack of substantive biochemical evidences on the allergenic proteins of sunflower pollen.

Role of sunflower pollen grain as airborne allergen was controversial. Due to its larger pollen size (dimension 40.2 x 37.9 μm^2^), sunflower pollen grains were thought not to fly in the air; causing only occupational allergy among florists and horticulture based workers. Studies on the workers of a sunflower processing industry also demonstrated that regular exposure to sunflower pollen grains often developed occupational allergic syndromes including severe lung impairment, allergic rhinitis and conjunctivitis [10]. However, the idea was changed when Jimenez et al. [11] reported frequent occurrence of sunflower pollen in ambient outdoor air. In this report the sunflower pollen grains were considered as potent aero-allergen for the individuals living in close vicinity of sunflower plantation. Therefore, identification of the immuno-reactive proteins from this pollen will facilitate understanding the molecular and immunological basis of sunflower pollinosis.

With the technical advancement in proteomics including mass spectrometry and bioinformatics, allergen identification has become more precise and accurate. It is now possible to detect and identify all the possible major and minor allergens by immunoscreening the single proteome of a particular source such as pollen, fungi, food and insect with sera from corresponding patients. Now-a-days mass spectrometry is the method of choice for allergen identification based on homology searching [12]. Some of the plant species from which pollen allergens have been identified and characterized by gel electrophoresis and mass spectrometry based proteomics are *Plantago lanceolata* [13], *Beta vulgaris* [14], *Betula verrucosa* [15] and *Artemisia vulgaris* [6]. However, little is known about sunflower pollen allergenicity. Till date only two allergens viz, Hel a 1 (34 kDa) [11] and Hel a 2 (14.7 kDa) [16] were primarily reported in IUIS database. Other works also reported the presence of three IgE reactive proteins in sunflower pollen [17]. However, comprehensive studies to understand the biochemical nature of the allergenic proteins from sunflower pollen are still in quest. Present study, to the best of our knowledge, is the first comprehensive study on the inhalant allergens of sunflower pollen using immunoproteomic approach.

The present study is conducted with the specific aim of identifying and characterizing the allergenic proteins from sunflower pollen grains using proteomic techniques. Allergenicity of *H*. *annuus* pollen grains were primarily investigated by clinical studies including in vivo and in vitro tests, such as, SPT, ELISA and histamine assay, followed by detailed immuno-biochemical and immunoproteomic analyses. In fact, this is the first report on the occurrence of sunflower pollen allergy in the Indian megacity of Kolkata.

## Materials and Methods

### Ethics statement

The present study protocol was approved by the human ethics committee of Bose Institute and Mediland Diagnostic Clinic, Kolkata. Informed written consents were obtained from patients and non-allergic volunteers for participation in the study. In case of minors, informed written consents were obtained from their guardians.

### Chemicals

All kits and reagents were purchased from Millipore Corporation (USA), Sigma-Aldrich (USA), GE-Healthcare (Sweden), GE Lifesciences (USA), G Bioscience (USA), Promega (USA), Merck (Germany), Bio-Rad (USA), Thermo Fischer(USA), Immunotech (France), Amersham (Sweden), Bruker Daltonics (Germany), Bruker Michrom (USA), Pathozyme (India), GeNei (India) and were of molecular biology grade.

### Pollen sampling

Pure pollen grains of *H*. *annuus* were collected from mature anthers of the fresh flowers growing around the city during their peak flowering period (April to first week of July of 2012–2014). The batch used throughout this work contained less than 1% of non pollen contaminants. 200 micron mesh was used to filter out unwanted debris.

### Allergen extract for Clinical Test (Skin Prick Test)

After defatting in diethyl ether, total pollen protein was extracted from 1 g of pollen in 20 ml of 0.1 M phosphate buffer (PB), pH 7.2 as described earlier [18]. Centrifugation was done at 12,000×g for 20 min to obtain the clear supernatant, which was passed through a 0.22 μm Millipore filter (Millipore Corp.) and used as antigen extract for pollen allergy diagnosis.

### Patient selection, Skin Prick Test (SPT), total IgE and histamine assay

Pollen allergic patients visiting Mediland Diagnostics, Kolkata were tested with antigenic extract of sunflower pollen grains. During SPT, 20 μL antigen extracts were placed on the ventral side of the forearm and the skin was pricked with a sterile lancet, without inducing bleeding. Histamine diphosphate (1 mg/mL) and PB (0.01 M, pH 7.2) were used as positive and negative controls, respectively. The wheal and flare reaction of the skin was read after 20 minutes by measuring wheal diameter and graded according to Platts-Mills et al [19]. Wheal diameter of ≥ 3 mm was recorded as positive response. The exclusion criteria for skin testing were pregnancy or lactation, malignancy and other severe systemic diseases. Corticosteroids and antihistamines were prohibited for 48 hrs before SPT, for the avoidance of decreasing sensitivity of SPT as these anti-allergic medications can inhibit allergic reactions in the skin. Patients with positive cutaneous response against sunflower pollen antigen were selected and 5ml of peripheral blood were collected for immunological studies. The patient group in our study was represented by 20 individuals sensitive to sunflower pollen, while the control group included 6 non-atopic subjects. The total IgE and released histamine in the sera were quantified by using commercial Total IgE quantification kit (Pathozyme) and EIA Histamine kit (Immunotech) respectively, following manufacturer’s protocol. All the serum samples were stored at −20°C.

### Sunflower specific IgE estimation

Indirect ELISA was performed to estimate the level of specific IgE in patient’s sera against crude antigenic extracts of pollen grains following the method as described earlier [20, 21]. Briefly, microtiter plates (Nunc, Thermo) were coated with crude antigen (100 ng/μl) of *Helianthus* pollen in carbonate-bicarbonate buffer (pH 9.2) and incubated overnight at 4°C. After repeated washing and blocking with 1% bovine serum albumin (BSA) (Sigma), wells were incubated with individual patients' sera (in 1:5 dilution) at 37°C for 16 h. Anti-human IgE tagged with alkaline phosphatase (1:1000 dilution) was used as secondary antibody. Colour was developed with para-Nitro Phenyl Phosphate (pNPP). The reaction was stopped by adding 3N NaOH and absorbance was measured at 405 nm. An individual serum having P/N value [ratio between mean OD_405_ of patient sera (P) and the healthy control (N)] more than 2.5 were considered as having high specific IgE titer and selected for further studies.

### Total protein profiling

Total pollen protein extracted in 20 ml of 0.1M PB, pH 7.2, was precipitated by salting out with 95% ammonium sulphate. Protein pellet was reconstituted in 4 ml of PB and dialyzed to remove traces of ammonium sulphate. Proteins (120 μg per well) were mixed with 20 μl of sodium dodecyl sulphate (SDS) loading buffer (0.125 M Tris–HCl pH 6.8, 5% SDS, 20% glycerol, 5% β-mercaptoethanol, 0.02% bromophenol blue), boiled for 5 min and run in 12% SDS-PAGE [22] using Mini-vertical gel electrophoresis apparatus (Amersham). The protein profile was visualized by CBB-R250 staining.

### IgE specific Western Blot

For immunoblot, resolved total protein was transferred onto polyvinylidene difluoride (PVDF) membrane (GE Lifesciences) by semi-dry transfer method as described elsewhere [21].The transfer was verified by reversible staining of the membrane with 0.1% PonceauS (w/v) in 5.0% acetic acid (w/v). Membrane was then rinsed with deionized water for 3h to completely remove PonceauS and cut into 10 mm strips. Membrane strips were blocked with 3% BSA in Tris buffer saline or TBS (50 mM Tris-Cl, 150mM NaCl, pH 7.5) containing 0.05% Tween-20 (TBS-T) at 4°C for 3 h. Following washing the strips were incubated overnight with 1:10 diluted sunflower pollen positive patients’ sera at 4°C. Twenty individual patient's sera with elevated level of specific IgE (as confirmed by ELISA) were used as primary antibody. Healthy serum pool was taken as negative control. Unbound IgE were removed by washing with TBS-T and strips were incubated with 1:1000 diluted secondary antibody, i.e., monoclonal anti-human IgE alkaline phosphatase conjugate (Sigma) at 4°C for 3h. Blots were devolpoed with nitro-blue tetrazolium -5-bromo-4-chloro-3'-indolyphosphate (NBT-BCIP) (Sigma). Finally the reaction was stopped by adding 0.5 mM ethylenediaminetetraacetic acid (EDTA).

### Protein extraction for proteome analysis

For proteomic analysis, total protein was extracted using trichloro-acetic acid (TCA)-acetone protocol following the method described earlier [23] with slight modifications. 100mg of *Helianthus* pollen was homogenized in liquid N_2_ and resuspended in 4 ml acetone containing 10% TCA plus 1% dithiothreitol (DTT) and incubated overnight at -20°C. After centrifugation at 15 000× gat 4°C for 30 min, the supernatant was removed and the pellet was rinsed thrice in ice-cold acetone with 1% DTT. The pellet was air-dried and resuspended in 100 μL rehydration buffer (IEF) containing 7M urea, 2M thiourea and 4%3-((3-cholamidopropyl) dimethylammonio)-1-propanesulfonate (CHAPS) (without DTT and ampholytes) and kept at -20°C for 16h. Finally it was centrifuged at 15 000× g at 4°C for 30 minutes; supernatant was collected and stored at -80°C. Protein concentration in the sample was quantified using Bradford reagent (BioRad).

### 2Dimensional (2D) gel electrophoresis

2D electrophoresis was performed following the protocol earlier described [24]with minor modification. Around 120μg of protein was processed thoroughly using Focus Perfect 2D Clean up Kit (G Biosciences), following manufacturer’s protocol. The clear protein was reconstituted in 125 μl rehydration buffer containing 0.75% IPG (pH 3–10 linear) buffer (v/v) (GE Healthcare), 25 mM DTT and traces of bromophenol blue. The sample was applied to 7 cm IPG strip (pH 3–10 Linear) in a re-swelling tray and left overnight at room temperature for rehydration. Separation of proteins in the first dimension was carried out in Ettan IPGphor 3 isoelectric focusing system (GE Lifescience) as per manufacturer’s instructions. Briefly, the strips were focused at 0–200 V for 1 min, 200–3500 V for 1.30 h and 3500 V for 1.15 h, with a total of 8 kVh accumulated. After focusing strips were equilibrated with equilibration buffer-I (6 M Urea, 75 mM Tris–Cl pH 8.8, 30% glycerol, 2% SDS and 1% w/v DTT) for 15 min followed by equilibration buffer-II (same as equilibration buffer-I with 2.5% w/v iodoacetamide instead of DTT). After equilibration, the strip was placed over 1 mm thick 12% vertical acrylamide gel and overlaid with molten 0.5% agarose sealing solution containing 0.002% (w/v) bromophenol blue dye. The second dimension separation was performed in miniVE Vertical Electrophoresis System (GE Healthcare). Gels were stained with Coomassie Brilliant Blue-R250 and photographed in a Bio-Rad Versa Doc (Bio-Rad Laboratories) system.

### 2D Immmunoblot and image analysis

Proteins from 7 cm 2D gel were subjected to semi dry transfer onto PVDF membrane and immuno- blotted as described previously in “IgE specific Western Blot”. After that, the PVDF membrane was blocked with BSA and then probed with sera collected from atopic patients. Twenty separate immunoblots were performed with 20 individual serum samples. In addition, a blot was also developed by confronting with pooled sera from 20 patients included in this study. Another blot was developed with sera from healthy subjects as negative control. Images of all the blots were acquired in Bio-Rad Versa Doc (Bio-Rad Laboratories) system and analyzed by Quantity One® software (version 4.6.3, Bio-Rad Laboratories).We considered only those sero-reactive spots with high signal (intensity value greater than 2).

### Periodic Acid Schiff (PAS) staining

Glycoproteins in the total protein were detected using Glycoprotein Staining Kit (G Bioscience) following manufacturer’s protocol. The kit uses an enhanced Periodic Acid‐Schiff (PAS) method for detection of sugar moiety. The supplied oxidizing agent in the kit first oxidized the cis‐diol groups of the aldehydes in sunflower glcoprotein, which then reacted with the Schiff reagent to produce strong magenta colored bands. RAPIDstain™ was used after glycoprotein staining to counter stain the non‐ glycosylated proteins.

### Deglycosylation study of allergens in 2D blot

Deglycosylation study was performed to remove the attached sugar moiety from glycoproteins through sodium metaperiodate treatment as described by Sircar et al. [21]. Briefly, proteins from 2D gel were transferred onto PVDF membrane and the membrane was incubated with 10 mM sodium acetate buffer (pH 5.0) containing 50 mM sodium metaperiodate for 3 h in the dark, after which the periodate was inactivated by adding a drop of ethylene glycol. Finally, the membrane was incubated with sodium borohydride solution (1 mg/ml) at 4°C for 12 h. The membrane was acidified by adding 0.001% acetic acid in borohydrate solution followed by repeated washing in TBS-T and then blocked with 3% BSA. IgE-western blotting was done with 1:10 diluted pooled patient sera in blocking solution.

### Hierarchical cluster analysis

A qualitative matrix designed on presence/absence of each allergen in all immunoblots was submitted to Hierarchical Clustering Analysis, processed with Complete Linkage clustering method according to the Euclidean distance using Spotfire TIBCO software, v.6.0. Clinical differences between classes were investigated through Dot Plot analysis using GraphPad Prism software, v.6.03.

### Sample preparation for mass spectrometry

For mass spectrometry (MALDI TOF/TOF and LC ESI qTOF), spots from 2D gel corresponding to the IgE reactive spots on 2D blot, were excised and subjected to in-gel trypsin digestion following the protocol as described by Shevchenko et al. [25] with slight modifications. Briefly, the gel pieces were destained with ethanol in 50mM ammonium bicarbonate (pH 8.0) (1:1 v/v) and Acetonitrile (ACN). Reduction and alkylation was done with 10mM DTT and 55mM iodoacetamide respectively. Digestion was carried out in 12.5 ng/μl modified sequencing grade Trypsin Gold (Promega) at 37°C for 16 h. Tryptic fragments were eluted from gel pieces by vigorous vortexing in extraction buffer containing 3% TFA and 30% ACN. Final volume of the sample was reduced up to 10 times in Speed Vac (Thermo Fischer). Approximately, 1.5 μl of peptide digests were mixed with 5 volumes of 0.5 mg/ml α-cyano-4-hydroxycinnamic acid (CHCA) matrix solution (Bruker Daltonics), spotted on MTP 384 ground steel target plate (Bruker Daltonics) and air dried. For LC-ESI experiments the solvent was completely evaporated and the peptides were dissolved in a suitable volume of 2% ACN containing 0.1% formic acid. This reconstituted sample was then mixed with solvent A used for loading onto LC-column.

### MALDI-TOF/TOF analysis

Mass spectra of trypsin digested proteins were obtained in Autoflex II MALDI TOF/TOF (Bruker Daltonics). Mass spectra were recorded in linear mode equipped with a pulsed N_2_ laser (λ = 337nm, 50 Hz) at 54% power in positive ion mode. After MS spectra acquisition, the instrument was switched to LIFT mode. The MS/MS spectra of top ten peptides with highest intensity were recorded by fragmentation of these peptides using LID (laser induced dissociation). MS/MS spectra were acquired with a minimum of 4000 and a maximum of 8000 laser shots using the instrument calibration file. Spectra baseline subtraction, smoothing [26] and centroiding were performed in FlexAnalysis software v3.0 (Bruker Daltonics).

### LC-ESI qTOF analysis

All the MS and MS/MS experiments for peptide identification were performed using a maXis impact™ high resolution qTOF mass spectrometer (Bruker Daltonics) equipped with a Captive Spray (Bruker Michrom) electrospray ionization platform. Separation was performed on a Dionex PepMap C18 column (250mmx75μm, 3 μm particle). The flow rate was set at 300 nL/min. The mobile phases A and B were 0.1% formic acid in water and 0.1% formic acid in 80% ACN, respectively. Positive ions (charge state +1, +2, +3) were generated by the electrospray ionization captive source. The following source settings were used for all subsequent data collection: Drying gas (nitrogen): 3 L/min, Dry temperature: 150°C, Capillary voltage: start at 1500–1700 V, decrease in 50 V steps until signal drops and add 300 V, End plate offset: 0 V.

Survey scans were acquired within a range from 200 to 2000 m/z. The spectrometer sequentially conducted MS/MS (in CID chamber) on the precursor ions (+2 and +3 charge state, excluding +1) detected in the full scan in data independent manner. MS/MS scans were conducted within a range from 25 to 2000 m/z. All MS and MS/MS raw data were acquired in.mgf format using Bruker’s ProteinScape™ software (version 3.0).

### Database search and allergen identification

The raw MS/MS spectra processed using MS Biotools™ 3.2(Bruker Daltonics) was used as input to an in house MASCOT search engine version 2.2 using the following criteria: minimum signal-to noise ratio: 20; peak density filter: 5 peaks per 200 Da and maximum number of peaks: 20. Searches were conducted with the following settings: one missed cleavage, P < 0.05 as significance threshold, the mass tolerance of precursor and fragment ions: 0.5 Da and 1.2 Da, respectively for MALDI TOF/TOF whereas 40 ppm and 100 ppm respectively for LC-ESI qTOF, carbamidomethylation of cysteine as fixed modification, methionine oxidation as variable modification, peptide charge: +1 for MALDI TOF/TOF and +2, +3, +4 for LC-ESI qTOF. Spectra were initially searched against NCBInr database, however unmatched peptides with low significance score were further subjected to similarity searches against the *H*. *annuus* EST database (downloaded from NCBI on June, 2015) containing 134474 entries. FASTA sequences of respective EST clones were analyzed by BLASTx algorithm, which searched for any signature sequence in the translated query to identify the protein.

## Result

### Determination of prevalence of allergenic sensitization to airborne sunflower pollen by SPT, specific IgE and histamine assay

In-vivo positive cutaneous reaction (SPT) towards sunflower pollen antigenic extract was observed among21%of the total pollen allergic patients (n = 354, age range: 16–50) screened in the present study. Among them +3 grade of positivity was found to be most predominant followed by +2 and +1. Here, we considered only +3 and +2 category patients (53 patients out of 74 sunflower pollen sensitized patients) and total thirty seven(22 of them with +3 grade) of such patients had given their written consent to use their blood for experimental purpose. The clinical and demographic features of the patients selected for serological analysis are presented in Table 1 and their clinical profiles of sensitization are included in S1 Table.

**Table 1:** Clinical features of the sunflower sensitized patient enrolled in the study.

Patient Number	Age	Sex	Symptoms*	SPT to sunflower (>3mm)**	Total IgE(kU_A_/L)	Specific IgE (P/N value)	Histamine content (nMol/L)
1	34	F	AR+ BA	+2	103	2.62	330.52
2	57	M	U	+3	124	3.56	355.91
3	48	F	AR+ ANG	+3	134	2.85	284.36
4	35	M	SOB	+3	100	3.69	306.97
5	25	F	CC	+2	119	3.86	370.53
6	24	F	CC	+3	122	4.26	367.92
7	20	F	AR+ BA+SOB	+3	127	4.09	370.29
8	16	M	SR	+2	105	2.77	207.76
9	30	F	CC	+3	105	3.43	309.03
10	55	F	AR+ BA	+3	107	2.59	255.71
11	47	M	SOB	+3	111	2.91	267.13
12	36	M	AR+ BA	+3	110	3.18	299.98
13	37	M	AR+ BA	+3	105	3.02	289.26
14	51	M	SR	+3	120	2.88	276.31
15	26	F	SR	+3	115	3.51	316.19
16	19	F	SR	+3	126	3.66	309.28
17	32	F	ANG	+3	121	3.37	305.32
18	34	M	CC	+2	116	2.71	269.23
19	26	F	CC	+2	112	3.45	257.84
20	59	M	ANG	+2	102	3.12	261.35
C1	18	M	NS	-	50	-	10
C2	20	F	NS	-	46	-	15
C3	35	M	NS	-	40	-	12.6
C4	30	F	NS	-	48	-	17.2
C5	59	M	NS	-	30	-	10.8
C6	55	F	NS	-	41	-	12

*Abbreviations: AR- Allergic Rhinitis, BA- Bronchial Asthma, SOB- Shortness of Breath, U- Urticaria, ANG- Angioedema, CC- Cough & Cold, SR- Skin Rash, NS- No Symptom, C1 –C6 –Non-atopic healthy subjects.

** The grading scale of positive skin prick reactions is as follows: negative if wheal diameter is <3 mm, +1 if wheal diameter is 3–5 mm, +2 if wheal diameter is >6 mm, +3 if wheal diameter is >6 mm, with one or two small pseudopodes, and +4 is any reaction that is more pronounced than +3.

In-vitro tests revealed significantly elevated levels of total IgE, sunflower specific IgE (P/N> 2.5) and histamine (>10nMol) in the sera of twenty patients as compared to healthy controls. Sera of these twenty patients were used in further immuno-biochemical studies for detail characterization of sunflower allergens.

### Detection of allergens by one dimensional IgE-immunoblot

**Fig 1:**
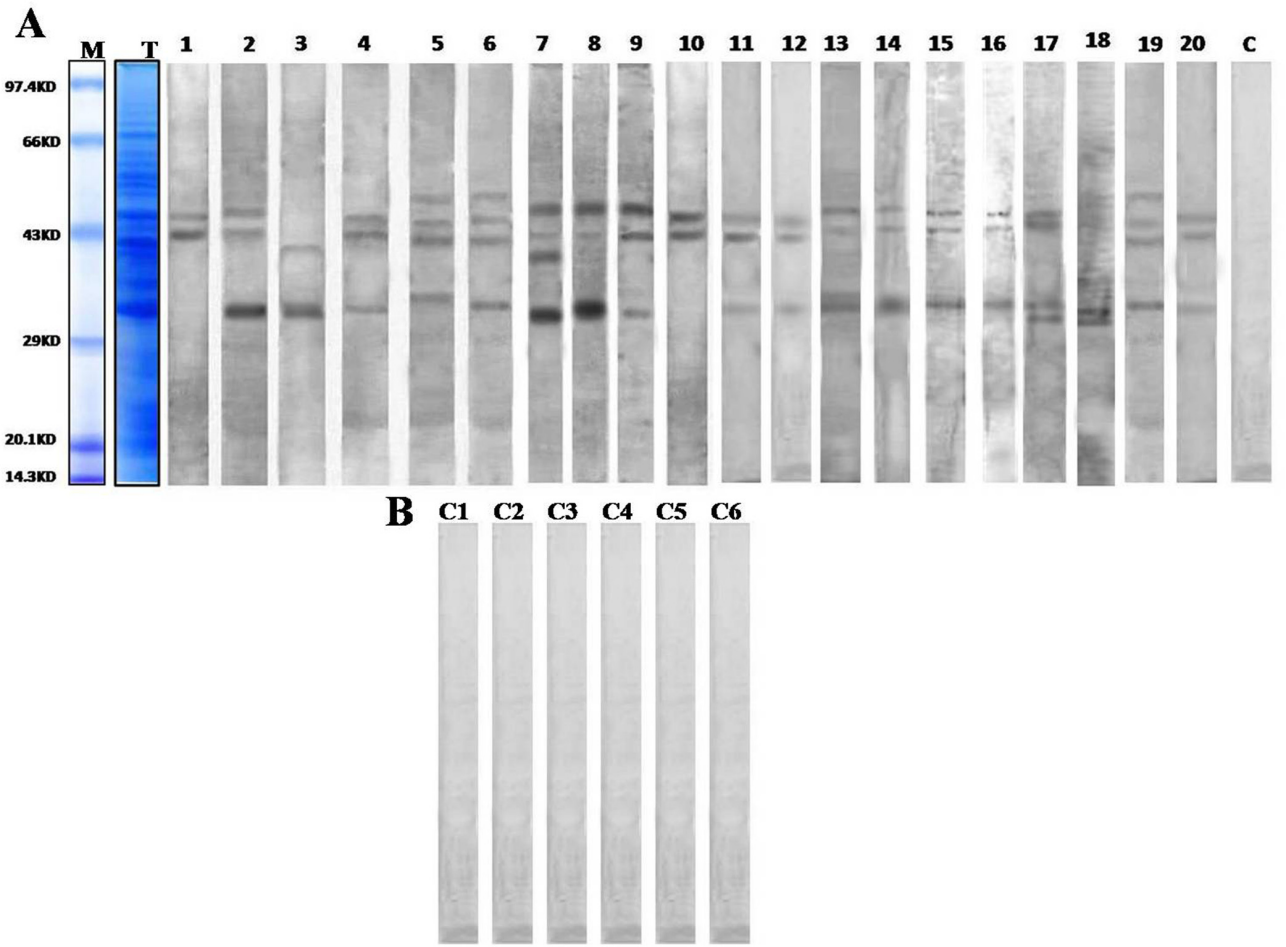
Total protein profiling and IgE-immunoscreening of allergens from sunflower pollen: A. Lane M: Medium range molecular weight marker, Lane T: Total protein profile of sunflower pollen in 12% SDS-PAGE as visualized by staining with CBB-R250, Lane 1–20: immunoblotting with individual serum of twenty sunflower positive atopic patients, Lane C: Negative control blot with pooled sera of non-atopic (healthy)subjects showing no IgE reactivity; **B**. Lane C1-C6: Negative control blot with individual sera of non-atopic (healthy) subjects showing no IgE reactivity

Total protein profile of sunflower pollen resolved in SDS-PAGE revealed more than forty coomassie stained protein bands within a MW range from14.3 to 97.4 kDa [Fig 1, Lane-T].The IgE binding proteins were detected by western blot of total pollen protein with twenty selected individual patient serum [Fig 1A, Lane 1–20]. This IgE immunoblot showed three distinct sero-reactive bands at MW of 39 kDa, 44 kDa and 49 kDa. In Addition, certain IgE reactive bands also appeared at MW regions of 32–34 kDa and 41–42 kDa. We considered these allergenic bands as two IgE reactive zones due to insufficient resolution of proteins particularly in these regions. Immunoblot with 6 non-atopic controls are also represented in Fig 1B.

### Sunflower pollen proteome profile in 2D gel and detection of IgE-reactive spots

**Fig 2:**
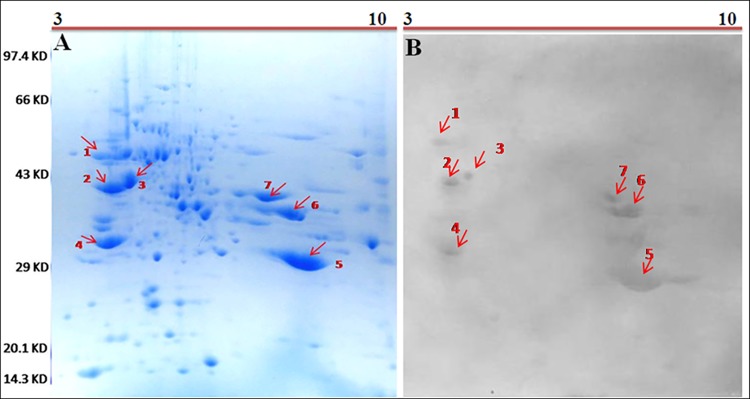
Two dimensional proteome mapping of sunflower pollen and detection of IgE reactive proteins by 2D-immunoblot: A. Proteome of sunflower *(H*. *annuus)* pollen in 2D gel within a pH range 3–10; B. 2D immunoblot with pooled sera from 20 atopic patients revealed seven distinct IgE reactive spots on 2D blot.

As illustrated in Fig 2A, the pollen proteome of sunflower in 2D gel was resolved into more than hundred spots within isoelectric point (pI) range of 3–10 horizontally and MW range of 14.3–97.4 kDa vertically. Fig 2B represents 2D immunoblot of the pollen proteome after being confronted with pooled sera from twenty sunflower positive allergy patients. We detected seven IgE reactive spots in the 2D blot. The MW’s of all the IgE reactive spots in 2D immunoblot were in agreement with that of the IgE reactive bands in 1D immunoblot.

#### Glycoprotein allergens and their IgE reactivity in 2D immunblot after deglycosylation

**Fig 3:**
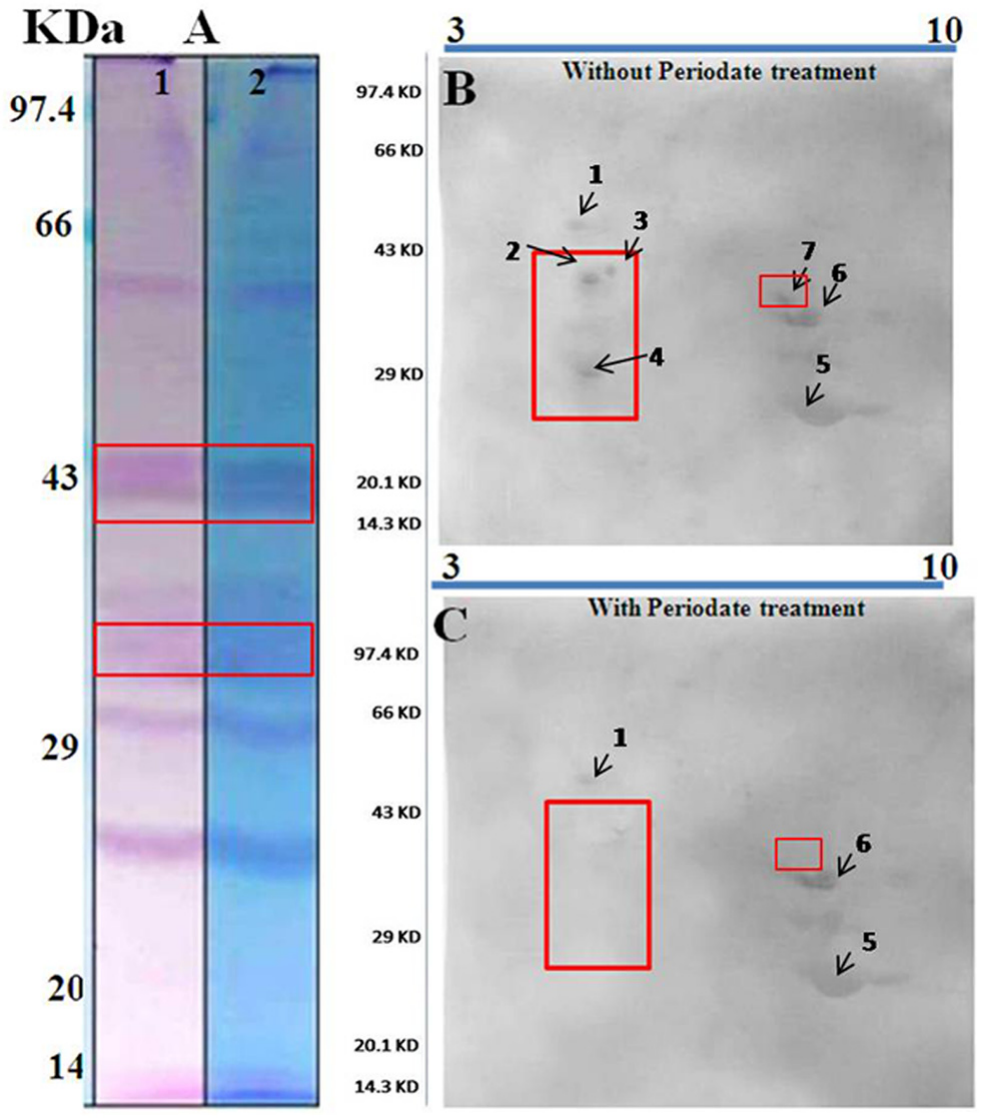
Detection of glycoprotein allergens in sunflower pollen and their antigenecity by metaperiodate mediated deglycosylation study in 2D blot: A. PAS staining of sunflower pollen protein in 1D SDS-PAGE to detect the glycoproteins, which turned into purple colors. The PAS stained gel was compared with IgE-immunoblot shown in Fig 1, which identified three glycoprotein regions as possible IgE reactive zones highlighted in boxes. Lane 1: sunflower pollen rotein in SDS-PAGE after PAS staining, Lane 2: Counter staining the PAS stained gel with CBB- G250 that turned only non-glycoproteins into blue. B & C. 2D immunoblot without metaperiodate treatment (B) was compared with another 2D immunoblot after metaperiodate (C) treatment. The metaperiodate treatment resulted in loss of IgE reactivity of certain spots (marked within box) suggesting the possible involvement of sugar moiety to determine the IgE binding of the allergens.

PAS staining was done to detect the possible glycoproteins from IgE-reactive molecules. Schiff staining of the total protein profile in SDS-PAGE showed magenta coloration of the bands near 88 kDa, 58 kDa, 42–44 kDa, 29–38 kDa and 26 kDa; hence, these were considered as glycoproteins [Fig 3A]. This result was compared with our previous immunoblot and it was found that the IgE-reactive bands of MW 41–42 kDa, 44 kDa and 32–34 kDa respectively, could be possible glycoproteins as these were present within PAS stained regions of SDS-PAGE. Remaining other IgE-reactive bands did not appear to be PAS stained.

Deglycosylation study followed by 2D-immunoblot was performed to understand the antigenic role of sugar moieties of the glycoprotein allergens. Meta periodate treatment of PVDF membrane destroyed the glycol-structures of the glycoproteins, which was then probed with pooled patient sera. The 2D immunoblot developed after deglycosylation was compared with non-deglycosylated 2D blot. As shown in Fig 3B and 3C, four sunflower pollen allergens (spot number 2, 3,4and 7) were found to have no IgE-reactivity after deglycosylation, suggesting their glycopeptidic nature of the IgE binding epitopes.

### Determination of major sunflower pollen allergen(s) by cluster analysis of patient’s sensitization profile

**Fig 4:**
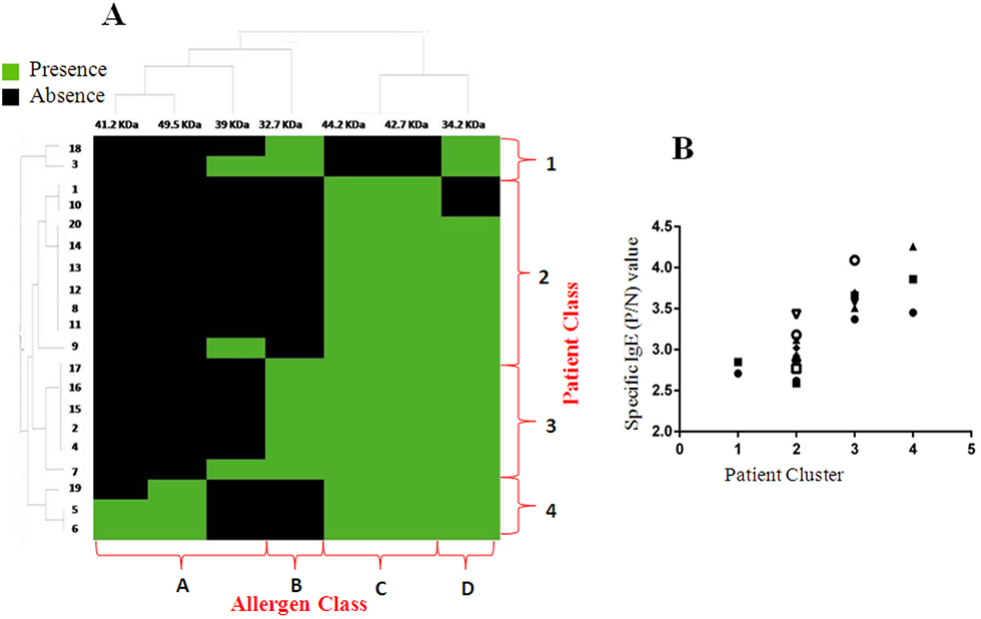
Hierarchical cluster analysis based on individual patient’s immunological profile and the corresponding immuno-reactive components: A. Heat map representing a qualitative summary matrix based on presence/absence of IgE-reactive components in 2D immunoblots of individual patient serum. The clustered data was processed with complete linkage according to Euclidean distance. Column clustering divided allergens into four classes (A- D) based on their frequency of IgE-reactivity. Row clustering divided patients into four classes (1–4) depending upon the sensitivity towards sunflower pollen allergen. B. Scatter plot analysis of sunflower specific IgE levels in serum pool of each of the four patient clusters generated by hierarchical cluster analysis. Each dot represents a single patient within a cluster.

A series of twenty different 2D immunoblots were performed with twenty individual patient sera to define the IgE reactivity profile of each patient towards sunflower pollen allergens. Cluster analysis was performed using a matrix of binary data based on presence/absence of IgE reactive proteins in each immunoblot (Fig 4A). Column clustering revealed four distinct classes (Class A to D) based on the frequency of IgE reactive spots in examined cohort. The constituent IgE-reactive spots in each of these four ‘column clusters’ are shown in Table 2. The algorithm also defined four major ‘row clusters’ (Class 1 to 4) based on IgE reactivity profile of each patient. The constituent patients in each of these four ‘row clusters’ are also listed in Table 2. Sensitivity profile of two patients in Class 1 showed predominant reactivity towards cluster B and D allergens. Class 2 was comparatively larger and composed of nine patients, who displayed reactivity towards Class C and D allergens. The only exception in Class 2 was patient number 1, who remained un-reactive to class D allergen. Sensitivity toward 39 kDa protein of Class A allergen was found only in patient number 9 of Class 2. Class 3 patients were characterized by their reactivity with Class B, C and D allergens. Class 4patients showed extensive reactivity to almost all sunflower pollen allergens. Only this cluster showed frequent reactivity to class A allergens in addition to Class B, C and D. Therefore, class 4 patients were found to be more susceptible to multiple sensitizations toward different inhalant allergens from sunflower pollen. This observation was further substantiated by the presence of more severe clinical symptoms, more complex immunoblot and significantly elevated level of sunflower specific IgE (Fig 4B) in the sera of class 4 patients. Representative 2D blots from each of the four Classes of patients are shown in S1 Fig.

**Table 2:** List of individual components within allergen class (column clustering) and patient class (row clustering) obtained by Hierarchical Cluster Analysis.

Column cluster		Row cluster	
Allergen class	IgE reactive spots	Patient class	Patient number
A	Spot no. 7 (41.2 KDa), Spot no. 1 (49.5KDa) and Spot no. 6 (39KDa)	1	18, 3
B	Spot no. 5 (32.7KDa)	2	1, 10, 20, 14, 13, 12, 8, 11 and 9
C	Spot no. 3 (44.2KDa) and spot no. 2 (42.7KDa)	3	17, 16, 15, 2, 4 and 7
D	Spot no. 4 (34.2KDa)	4	19, 5 and 6

From this hierarchical cluster analysis, it is also evident that Class C and D allergens were predominant among 95% of sunflower sensitive subjects. Thus we identified these as immunodominant major allergens from sunflower (*Helianthus annuus*) pollen.

### Mass spectrometry based identification of allergens

**Fig 5:**
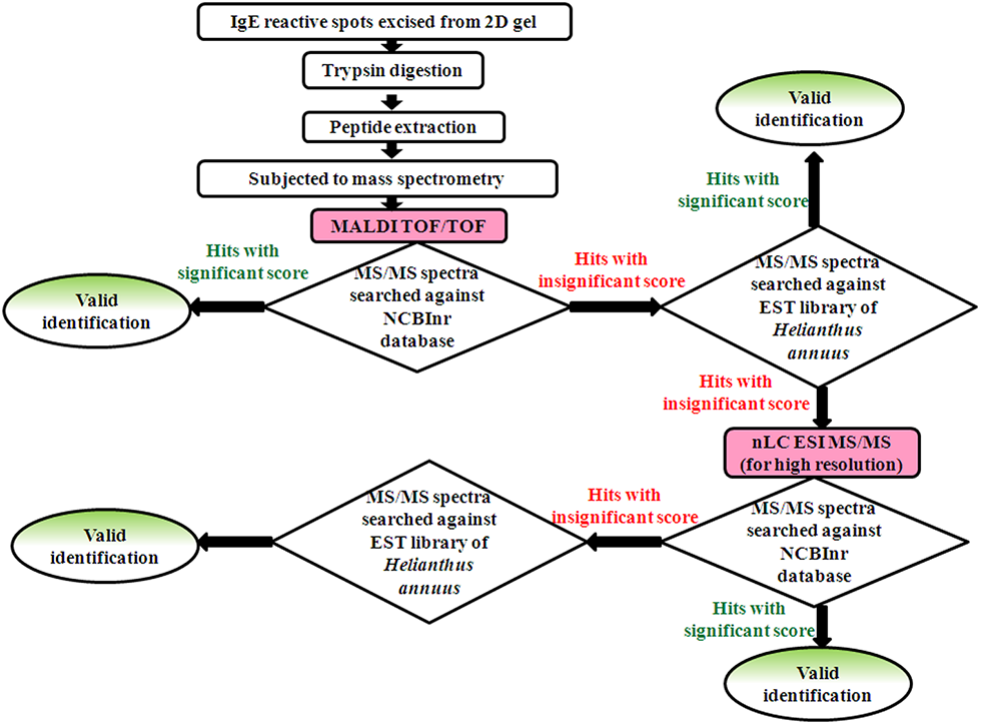
A schematic representation illustrating the mass spectrometry workflow adopted in the present study for proteomic identification of sunflower.

We have identified 7 IgE reactive proteins from sunflower pollen using a gel based layered proteomic approach by employing combinations of two different mass spectrometers (MALDI and ESI) and two different databases (NCBInr and sunflower EST collection)as illustrated in Fig 5. The major criteria for valid identification of proteins was based on the occurrence of at least two unique peptides with significance level p<0.05.

All the seven IgE reactive spots were excised from 2D gel, trypsin digested and subjected to MALDI-TOF/TOF. Four allergens (spot number 2, 4, 6 and 7) were identified with significant scores by MALDI-TOF/TOF. Except for spot number 2, which was successfully identified in NCBInr database, the other three spots displayed low confidence of identification. Hence, MALDI generated MS/MS spectra of these three spots were searched against EST library of *Helianthus annuus*. This resulted in significant homology for spot number 4, 6 and 7 with selected sunflower EST clones.

Three remaining spots (spot number 1, 3 and 5) which could not be identified by MALDI-TOF/TOF were subjected to LC-ESI qTOF. Searching of these MS/MS data against NCBInr database resulted in low confident protein identification. Hence the mass spectra were analyzed against EST library of *Helianthus annuus* as previously described for MALDI data, which led to successful identification of these three spots.

In nutshell, the mass spectrometry analysis identified six sunflower allergens (spot number 1 and 3 to 7) from the corresponding EST library whereas only one allergen (spot number 2) was identified from NCBInr database. In case of spot number 2, two peptides “R.WGTYAIGGSSAPTILSQGNR.F” and “R.FGFFQVVNNNYDR.W” were matched with pectate lyase amb a1.2 from *Ambrosia artemisiifolia* with peptide sequence coverage of 5.52% and 3.76% respectively. Three sunflower pollen proteins which came out to be as the major allergens in our cluster analysis were identified as pectate lyase (spot number 2 and 3) and cysteine protease (spot number 4) respectively. Detail results of mass spectrometry identification of sunflower pollen allergens are described in Table 3 and BLASTx analysis of EST clones are illustrated in Table 4.

**Table 3:** Mass spectrometry based identification of Sunflower (*Helianthus annuus*) pollen allergens.

Spot No.	Mass Spectrometer and Database Used	NCBI Accession No.	Protein Name (Organism)	No. of Unique Matched Peptides	Sequence of Unique Peptides	Significance Score for Peptide Identification (E Value)*	Gel Mass (kDa)/pI	Biological Function
1	ESI-qTOF and EST library	gi|90464401	Beta-tubulin (*Nicotiana attenuata*)	11	R.YLTASAMFR.G, K.IREEFPDR.M, R.YLTASAMFR.G, K.NMMCAADPR.H, K.LAVNLIPFPR.L, R.FPGQLNSDLR.K, R.VSEQFTAMFR.R, K.SSVCDIPPTGMK.M, K.EVDEQMINVQNK.N, R.LHFFMVGFAPLTSR.G, K.MSSTFVGNSTSIQEMFK.R	0.00085, 0.084, 0.27, 43, 0.0001, 2.9e-007, 0.051, 0.0054, 2.2e-005, 0.02, 1.2e-009	49.5 kDa /4.75	GTPase
2	MALDI TOF/TOF and NCBInr	gi|113476	Pectate lyase I, Pollen allergen Amb a 1.2 (*Ambrosia artimisiifolia*)	2	R.WGTYAIGGSSAPTILSQGNR.F, R.FGFFQVVNNNYDR.W	0.00012, 0.21	42.7 kDa/4.91	Pectin degradation
3	ESI-qTOF and EST library	gi|90459099	Putative pectate lyase precursor (*Ambrosia artimisiifolia*)	6	K.ADWADNR.Q, R.FLAPDDAAK.K, R.QAMADCAQGFAK.G, K.QIWIDHCSFSK.A, K.VMLLGADDGHHQDK.N, R.WGTYAIGGSSAPTILSQGNR.F	0.017, 0.00098, 1.1e-005, 5.2e-005, 7.3e-007, 3.6e-006	44.2kDa/5.39	Pectin degradation
4	MALDI TOF/TOF and EST library	gi|90442509	Cysteine protease (*Ambrosia artimisiifolia*)	2	R.TGQLLSLSEQQLLDCDSSDR.T, K.NQGQCGSCFAFAAVGAIEGINAIR.T	6e-007, 1.10E-07	34.2 kDa /4.909	Peptidase
5	ESI-qTOF and EST library	gi|90455367	NtPRp27-like, partial (*Senecio aethnensis*)	2	K.IIGGVPFTK.K, K.TPIPLVEGIABYTILK.A	0.013, 4.2e-007	32.7 kDa /8.11	Pathogenesis related proteins
6	MALDI TOF/TOF and EST library	gi|22389921	Glyceraldehyde-3-phosphate dehydrogenase, putative (*Ricinus communis*)	1	R.VPTVDVSVVDLTAR.L	0.04	37.8 kDa /8.11	Glyceraldehyde-3-phosphate dehydrogenase catalyze the conversion of Glyceraldehyde-3-phosphate to 1,3-bisphosphoglycerate
		gi|90454328	Glyceraldehyde-3-phosphate dehydrogenase C subunit (*Gossypium hirsutum*)	1	K.LVSWYDNEWGYSNR.V	0.27	37.8 kDa /8.11	Glyceraldehyde-3-phosphate dehydrogenase catalyze the conversion of Glyceraldehyde-3-phosphate to 1,3-bisphosphoglycerate
7	MALDI TOF/TOF and EST library	gi|90461212	Predicted fructose-bisphosphate aldolase cytoplasmic isozyme-like (*Citrus sinensis*)	2	K.VAPEVVGEYTVR.A, K.IGPNEPSPLSIMENAYGLAR.Y	0.26, 0.36	41.2 kDa /7.77	Aldolase, glycolytic enzyme

*All the scores are as significant as p< 0.05.

**Table 4:** BLASTx search analysis using sunflower EST clones (identified in MS/MS) as input against NCBInr database.

Spot No.	NCBI Accession No. of Sunflower EST	Protein hits in BLASTx (Organism)	NCBI Accession No. of matched proteins	Query coverage#	E value##	Sequence Identity###
1	gi|90464401	Beta-tubulin (*Nicotiana attenuata*)	gi|40036995	88%	0	97%
3	gi|90459099	Putative pectate lyase precursor (*Ambrosia artimisiifolia*)	gi|302127816	95%	5.00E-146	80%
4	gi|90442509	Cysteine protease (*Ambrosia artimisiifolia*)	gi|558482540	98%	3.00E-96	54%
5	gi|90455367	NtPRp27-like, partial (*Senecio aethnensis*)	gi|409189751	74%	1.00E-72	55%
6	gi|22389921	Glyceraldehyde-3-phosphate dehydrogenase, putative (*Ricinus communis*)	gi|255544534	65%	4.00E-65	94%
	gi|90454328	Glyceraldehyde-3-phosphate dehydrogenase C subunit (*Gossypium hirsutum*)	gi|211906518	92%	3.00E-135	91%
7	CHAY6293.b1_J13.ab1 CHA(XYZ)	Predicted: fructose-bisphosphate aldolase cytoplasmic isozyme-like (*Citrus sinensis*)	gi|568856629	94%	2.00E-165	89%

#: Query coverage

##: E value

###: and sequence identity were calculated for matched proteins in NCBI database with respect to each corresponding EST clones.

## Discussion

Allergic diseases associated with IgE mediated sensitization to Compositae pollen allergens has been increasing at an alarming rate worldwide with India being no exception. With the increase in sunflower plantation for agro-commercial purpose, allergenicity to its pollen grains has now become a serious health concerns for patients suffering from respiratory allergic disorder. Lack of information on allergenic components in sunflower pollen has rendered the clinical diagnosis and treatment of sunflower pollinosis largely inexplicable. Here we present a detail clinical, immuno-biochemical and proteomic approach with an aim to precisely characterize the inhalant allergens of sunflower pollen.

### Sunflower pollen causes IgE-mediated atopic sensitization of respiratory tract

The present study started with clinical investigation on the predominance of sunflower pollen allergy among the atopic population of Kolkata megacity of India. A major part of the allergy sufferers (20.62%) were diagnosed to have atopic sensitivity towards sunflower pollen antigens. In these patients, the atopic sensitivity together with respiratory allergic inflammation was found to have been associated with the presence of serum IgE specific for sunflower pollen extract and histamines, as determined by in-vitro confirmatory tests. Whereas the earlier published literatures on ‘sunflower allergy’[10, 11, 16, 17] mainly predicted a close association between the occurrences of sunflower pollen in the ambient outdoor air and prevalence of respiratory allergic symptoms, our present study has indeed strengthened this notion by adding new sunflower allergens to the meager list of Asteraceae pollen allergens reported so far.

### Sunflower pollen proteome is a repertoire of inhalant allergens

In order to explore the sero-reactive proteins from the pollen proteome of sunflower, an allergenomic study was undertaken. This study was basically a discovery approach based on one dimension and two dimensional separation of pollen proteins followed by immunoscreening of allergens using sera from sunflower pollen sensitive patients. Multiple IgE reactive bands observed in 1D immunoblot against individual patient serum suggested sunflower pollen as a source of different allergens.

A major limitation of 1D immunoblot is insufficient resolution of proteins rendering the precise identification of these allergens quite difficult. Hence, we have taken the help of 2D gel based proteomic tools, which are customarily used for better separation of allergens[27]. Five IgE reactive bands in 1D gel were found to resolve into seven distinct IgE reactive spots in 2D gel using the same set of sera. Thus 2D separation of sunflower pollen allergens has increased the chance of finding individual species in each excised spot and subsequent analysis by mass spectrometry.

### Sugar moieties of certain glycoprotein allergens of sunflower pollen are important for IgE-binding

Studies had shown that IgE binding glycoproteins are widespread among pollen grains [28]. Certain Asteraceae species for example mugwort (Art v 1) [29] and ragweed (Amb a 4) [30] were reported to produce pollen containing such allergens with a bulky arabinogalactan-protein moiety. These findings prompted us to investigate for the presence of any such glycoprotein allergens in sunflower, which is another member of Asteraceae family. PAS staining of sunflower pollen proteins in 1D SDS-PAGE revealed three regions of glycoproteins that closely resemble with the IgE reactive zones in immunoblot. One of these regions was in agreement with a 55 kDa glycoprotein allergen from sunflower pollen already reported by Hoz et al.[17]. In our study, we tried to determine the antigenic role of the glycan moiety by meta-periodate modification test in 2D blot. Loss of IgE reactivity ascertained the role of glycan part in eliciting IgE response whereas retention of IgE reactivity revealed the peptidic nature of IgE epitopes. In our case, four glycoprotein allergens of sunflower such as 44.2 kDa, 42.7 kDa, 34.2 kDa and 41.2 kDa were found to have their carbohydrate part acting as antigenic determinant.

### The major allergens of sunflower pollen are glycoproteins

Most interestingly the three glycoprotein allergens (44.2 kDa, 42.7 kDa and 34.2 kDa) were identified as major allergens of sunflower pollen since 95% of sunflower allergic patients were sensitive to these allergens as revealed by comparative cluster analysis. Among these three allergens 44.2 kDa and 42.7 kDa allergens constituted class C allergens and 34.2 kDa allergen constituted class D allergens. All these three allergens were frequently reacted with serum IgE of most of the patients in individual 2D blot. Alternatively, row clustering discriminated a distinct class of patients (Class 4) which showed extensive sensitization toward a larger variety of sunflower allergens. This class of patients is more susceptible to multiple sensitizations toward different allergen sources due to cross IgE reactivity.

### Novel allergens identified from sunflower pollen by mass spectrometry

In this study, all the IgE reactive spots detected from 2D proteome map of sunflower pollen were successfully identified using ‘bottom-up’ mass spectrometry. A major pitfall in proteomic studies on sunflower (*Helianthus annuus*) is the lack of its functional genome information, which hinders confident identification of proteins from the non-redundant databases (NCBI and Uniprot) using MS/MS data. Although most of the plant genomes are largely unsequenced due to extensiveness of the task, a number of EST sequences have been assembled in NCBI database to provide researchers some transcriptomic sequence data [31]. CGPDB (http://cgpdb.ucdavis.edu) is an EST sequencing project that offers 134474 online available EST clones (as on June, 2015) for *Helianthus annuus* to overcome the problem of lack of full annotation to some extent. We have efficiently utilized these sunflower EST data as reference database to search the MS/MS data. In the starting, MALDI-TOF/TOF was used to generate mass spectral data. However, for certain spots MALDI-TOF/TOF generated data were unable to find significant hit in any of the databases used in our study. Hence, for better peak resolution, maximum signal and maximum coverage, we used LC-MS/MS for those unassigned spots. This approach has enabled us to identify all the allergens without any ambiguity. Both spot number 2 and 3 showed homology with pectate lyase of *Ambrosia artemisiifolia*. Pectate lyase is an enzyme involved in pectin degradation during pollen tube growth [32]. Five isoforms of pectate lyase allergen have been reported from *Ambrosia artemisiifolia* (Amb a 1.1 to Amb a 1.5) of varying MW ranging from 42.709 kDa to 44.082 kDa and pI value from 5.58 to 6.63 [33]. Pectate lyase belongs to an allergen family (accession number: AF073) enlisting ten allergens reported so far, two of which, Amb a 1 and Art v 6 [34] were from Asteraceae family. Moreover, spot number 2 and 3 exhibited exactly same pattern of IgE-sensitization among the tested patients.

Spot number 4 (MW- 34.2 kDa) has been identified as another major allergen from sunflower pollen, displaying extensive reactivity almost across the entire patient groups, except one (patient number 3). The mass spectra of this spot upon searching against sunflower EST database displayed significant match with an EST clone (Gene bank acc.: DY904392), which partially represents a plant cysteine protease as revealed by BLASTx search. Proteases have been documented as important group of inhalant and food allergens. An already reported cysteine protease allergen is Amb a 11from ragweed pollen (*Ambrosia artemisiifolia*) [35].In addition to IgE reactivity, these allergens, by virtue of their protease activity can desquamate lung epithelial tissue to facilitate easy invasion of the allergens. All the eleven unique peptides of spot number 1 displayed homology with an EST clone (Genebank acc.: DY926280) which contains a putative conserved domain of tubulin FtsZ superfamily. Constitutively expressed tubulin protein was earlier reported as allergen from dust mites [36]. Our study is perhaps the first report on a plant specific tubulin as respiratory allergen from pollen grains.

In similar way, spot number 5 was identified as a putative member of GluZincin super family. It contains a highly conserved zinc binding metalloprotease (M36) domain with HEXXH and EXXXD motifs [37]. Such metalloproteases have been reported as allergen from certain pathogenic molds such as *Aspergillus fumigatus* (Asp f 5) [38] and are popularly known as fungalysins. These fungalysins are thought to aid in degradation of host lung cell walls during allergy and infection [38]. In case of plants, similar type of protein was found to get induced during biotic stress and thus considered as a pathogenesis related protein (PR-17 family) [39]. However, no previous reports exist that support the allergenic nature of this protein of plant origin. Here, we have identified a putative form of this metalloprotease as a novel allergen from sunflower pollen. Two unique peptides generated from trypsinization of 37 kDa spot number 6 had shown significant match with plant glyceraldehyde-3-phosphate dehydrogenase. This constitutively expressed glycolytic enzyme is a well known allergen from wheat as food allergen [40] and *Asprgillus versicolor* as indoor inhalant allergen [41].Spot number 7 has been identified as a fructose bisphosphate aldolase (FBPA) with two unique peptides. Until now only one class-II FBPA has been reported as allergen from skin colonizing *Candida albicans* [42]. The class-II FBPA are mainly prevalent among the prokaryotes and molds. In our study, the exact nature of sunflower FBPA could not be determined due to the corresponding incomplete EST sequence.

Taken together the entire mass spectrometry results, present study essentially describes a panel of IgE reactive molecules from sunflower pollen proteome by homology driven proteomics. Although similar types of allergens have already been discovered from other sources, still to the best of our knowledge, we report these proteins for the first time as novel allergens from sunflower pollen.

## Conclusion

Our present study is the pioneer report on the clinical evidence of sunflower pollen allergens as major threat for respiratory allergy patients, which was further corroborated by detail immuno-proteomic analysis. It also describes a useful strategy of mass spectrometry analysis involving two different ionization methods and two different database searches in order to fruitfully identify the allergens from plant species without any prior information on its genome sequence. Such strategy can also be effectively applied for other wildly grown weed species for which no genome information is available. The present study has primarily identified a total seven new sero-reactive allergens from sunflower pollen, three (two pectate lyase and a cysteine protease) of which have been detected as major and immunodominant sensitizers. These three major allergens were glycoproteins and most significantly, their glycan structures were found to take part in IgE binding.

Further studies such as purification, recombinant expression and epitope mapping of these allergens will open up new avenues to improve the current tools of component resolved diagnosis and immunotherapy of pollen allergy.

## Supporting Information

S1 Fig Representative 2D blot of individual sera from each of the four patient classes. The IgE reactive components appeared in twenty different 2D blots developed with twenty individual sera was used for cluster analysis to identify the most frequently reactive sunflower allergens as well as the most sensitive class of patients.
(TIF)

S1 Table Clinical profile of allergenicity (as investigated by SPT) of selected sunflower pollen allergic patients (DOCX)
