# Supplementary figures and images for: Search for Allergens from the Pollen Proteome of Sunflower (Helianthus annuus L.): A Major Sensitizer for Respiratory Allergy Patients
Source: PLoS One. 2015 Sep 29;10(9):e0138992. doi: 10.1371/journal.pone.0138992 (PMC4587886; doi:10.1371/journal.pone.0138992)

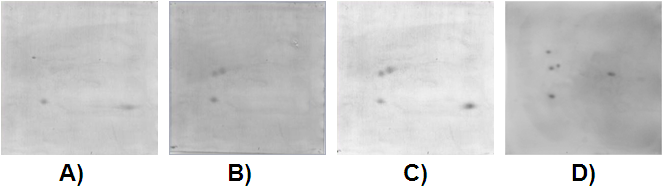

Supplement: S1 Fig — The IgE reactive components appeared in twenty different 2D blots developed with twenty individual sera was used for cluster analysis to identify the most frequently reactive sunflower allergens as well as the most sensitive class of patients. (TIF) [file pone.0138992.s001.tif]
