# Supplementary material for: Search for Allergens from the Pollen Proteome of Sunflower (Helianthus annuus L.): A Major Sensitizer for Respiratory Allergy Patients
Source: PLoS One. 2015 Sep 29;10(9):e0138992. doi: 10.1371/journal.pone.0138992 (PMC4587886; doi:10.1371/journal.pone.0138992)
Supplement: S1 Table — (DOCX) [file pone.0138992.s002.docx]

**S1 Table: Clinical profile of sensitization (as investigated by SPT) of selected sunflower pollen sensitized patients**

| **Patient number** | **SPT to different pollen grains** | | | | | | | | | **SPT to different fungal spores** | | | **SPT to different foodstuffs** | | | | | | | |
| --- | --- | --- | --- | --- | --- | --- | --- | --- | --- | --- | --- | --- | --- | --- | --- | --- | --- | --- | --- | --- |
|  | **Artemisia vulgaris** | **Parthenium hysterophorus** | **Cynodon dactylon** | **Cyperus rotundus** | **Cocos nucifera** | **Phoenix sylvestris** | **Carica papaya** | **Peltophorum pterocarpum** | **Eucalyptus citriodora** | **Aspergillus sp.** | **Penicillium sp.** | **Rhizopus oryzae** | **Egg** | **Milk** | **Wheat** | **Fruit** | | | | |
|  |  |  |  |  |  |  |  |  |  |  |  |  |  |  |  | **Apple** | **Banana** | **Orange** | **Litchi** | **Pear** |
| 1 | +1 | +2 | +2 | +2 | +2 | +1 | +1 | +1 | +1 | +1 | - | - | +1 | - | - | - | - | - | - | - |
| 2 | +3 | +3 | +2 | +1 | +3 | +2 | +1 | +2 | +2 | - | - | - | +1 | +1 | - | - | - | - | - | - |
| 3 | +2 | +2 | +2 | +1 | +1 | +1 | +2 | +1 | +1 | - | - | - | +1 | - | +1 | +2 | - | - | - | - |
| 4 | +3 | +3 | +1 | +2 | +3 | +3 | +1 | +3 | +1 | - | - | +1 | - | - | - | - | +2 | - | - | - |
| 5 | +3 | +3 | +2 | +3 | +2 | +2 | +2 | +2 | +2 | - | - | - | - | - | +1 | - | - | - | - | - |
| 6 | +3 | +2 | +2 | +2 | +2 | +3 | +2 | +3 | +2 | - | +1 | - | +1 | - | +1 | - | - | - | - | +1 |
| 7 | +3 | +3 | +2 | +2 | +2 | +2 | +2 | +3 | +2 | - | - | - | +1 | +1 | - | - | - | - | - | - |
| 8 | +2 | +1 | +2 | +1 | +1 | +1 | +1 | +2 | +1 | - | - | - | - | - | +1 | - | - | - | - | - |
| 9 | +1 | +2 | +2 | +3 | +2 | +1 | +1 | +1 | +3 | +1 | - | - | +1 | - | - | - | - | - | - | - |
| 10 | +2 | +3 | +2 | +1 | +2 | +2 | +1 | +1 | +1 | - | +1 | - | +1 | +1 | - | - | - | - | - | - |
| 11 | +2 | +3 | +2 | +1 | +2 | +1 | +3 | +1 | +1 | - | - | - | +1 | +1 | - | - | - | - | - | - |
| 12 | +2 | +2 | +2 | +3 | +2 | +1 | +1 | +1 | +1 | +1 | - | - | +1 | - | - | - | - | - | - | - |
| 13 | +3 | +3 | +2 | +1 | +2 | +3 | +1 | +1 | +1 | - | - | +1 | +1 | +1 | - | - | - | - | - | - |
| 14 | +3 | +2 | +2 | +1 | +1 | +1 | +2 | +1 | +1 | - | - | - | +1 | - | +1 | - | - | +2 | - | - |
| 15 | +2 | +2 | +2 | +3 | +2 | +1 | +1 | +1 | +1 | +1 | - | - | +1 | - | - | - | - | - | - | - |
| 16 | +3 | +2 | +2 | +1 | +1 | +1 | +2 | +1 | +1 | - | - | - | +1 | - | +1 | - | - | - | +2 | - |
| 17 | +2 | +3 | +2 | +1 | +2 | +2 | +1 | +2 | +2 | - | - | - | +1 | +1 | - | - | - | - | - | - |
| 18 | +1 | +1 | +2 | +1 | +1 | +1 | +1 | +1 | +1 | - | +1 | - | - | - | +1 | - | - | - | - | - |
| 19 | +3 | +3 | +2 | +2 | +2 | +2 | +3 | +3 | +2 | - | - | - | - | - | +1 | - | - | - | - | - |
| 20 | +2 | +1 | +2 | +1 | +1 | +1 | +1 | +1 | +1 | - | - | - | - | - | +1 | - | - | - | - | - |
